# The Prevalence of Accommodative and Binocular Dysfunctions in Children with Reading Difficulties

**DOI:** 10.3390/life15010007

**Published:** 2024-12-25

**Authors:** Ilze Ceple, Aiga Svede, Evita Serpa, Evita Kassaliete, Liva Volberga, Rita Mikelsone, Asnate Berzina, Angelina Ganebnaya, Linda Krauze, Gunta Krumina

**Affiliations:** Department of Optometry and Vision Science, Faculty of Science and Technology, University of Latvia, Jelgavas Street 1, LV-1004 Riga, Latvia; aiga.svede@lu.lv (A.S.); evita.serpa@lu.lv (E.S.); evita.kassaliete@lu.lv (E.K.); angelina.ganebnaya@lu.lv (A.G.); linda.krauze@lu.lv (L.K.); gunta.krumina@lu.lv (G.K.)

**Keywords:** reading difficulties, visual complaints, ocular complaints, refractive error, accommodative dysfunctions, binocular dysfunctions

## Abstract

Uncorrected refractive error and unsatisfactory performance on several clinical accommodation and binocular vision tests are more common in children who struggle with reading. The aim of the current study is to explore the prevalence of accommodative and binocular dysfunctions in children with and without reading difficulties. Reading performance was assessed with the Acadience Reading (formerly DIBELS Next) test adjusted and validated for the Latvian language. Children with (N = 39) and without (N = 43) reading difficulties underwent thorough assessment of their subjective refraction, as well as binocular and accommodation functions. The results demonstrate no difference in the prevalence of complaints between children with and without reading difficulties (26% and 23%, respectively). However, children with reading difficulties more frequently present with significant uncorrected refractive errors and/or accommodative and binocular dysfunctions than children without reading difficulties (69% and 47%, respectively). According to the findings, even in cases where a child does not exhibit any ocular or visual complaints, a comprehensive visual function evaluation should be required for any child who struggles with reading.

## 1. Introduction

The human visual system plays a crucial role in the perception of the surrounding environment. It provides an enormous amount of visual information that we learn to process, integrate, and structure and is therefore involved in the processes of spatial orientation, human–human interaction, and other everyday activities [1,2,3]. The quality of one’s vision can also affect self-reported quality of life and psychological well-being, as well as potentially being related to academic achievement [4,5,6]. Glewwe et al. [7] demonstrated that by correcting refractive error in rural students, a significant improvement in academic performance could be observed. Similar results were obtained by Ma et al. [8], demonstrating a significant improvement in mathematics test scores as a result of an early vision screening and refraction correction. However, we have to keep in mind that an accurate performance of the visual system does not only rely on the correction of refractive error and good visual acuity, but also on a precise and coordinated performance of the accommodation and vergence systems.

After exploring the visual symptomology associated with refractive, accommodative, and non-strabismic binocular dysfunctions, Cacho-Martínez et al. [9] reported that patients with an uncorrected refractive error usually complained of headache, visual fatigue, blurred vision, eye dryness, sensitivity to light, sore eyes, and ocular pain. Some of the patients even reported avoiding near-vision tasks. Patients with accommodative dysfunctions mainly complain of red and sore eyes, visual fatigue, and difficulties in performing schoolwork [9]. Finally, patients with non-strabismic binocular dysfunctions mainly present with headache, visual fatigue, dry eyes, ocular pain, blurred vision, and lack of concentration. Although there may be some differences in complaints for different vision dysfunctions, in real life the difference is negligible, making it impossible to specify a child’s vision problem by looking at the complaints. The results of different questionnaires related to accommodative and non-strabismic binocular dysfunctions have demonstrated that a relatively high proportion of school-age children report subjective symptoms of headache, asthenopia, floating text, and facility problems [10,11]. An even more surprising observation by Junghans et al. [10] is that children referred to as the “emmetropic group” presented with more complaints related to near-vision work activities than children with previously diagnosed binocular vision dysfunctions.

The summary reports of child vision screening in Europe [12,13] indicate that in all EU countries, preschoolers (3–7 years old) are subjected to at least one vision screening, performed mainly by a general practitioner, orthoptist, ophthalmologist, optometrist, optician, nurse, or pediatrician. However, in most of the EU countries, school-age children are not obligated to perform any vision screening, and the management of refractive errors and accommodative and non-strabismic binocular dysfunctions depends on the recognition of different symptoms by the child, parents, teachers, doctors, or other surrounding persons [12]. In some cases, an abnormal head posture might be the only noticeable indicator of visual system dysfunctions [14]. Therefore, if the child is not asked specifically to identify some visual complaints, in some situations, the visual disorder might be unnoticed, and the reduced academic performance might be attributed to disinterest, laziness, or even serious learning problems (e.g., reading difficulties).

Children with reading difficulties are more likely to have an uncorrected refractive error and abnormal performance in different clinical accommodation and binocular vision tests. Two studies by Palomo-Álvarez and Puell [15,16] have demonstrated that children with poor reading skills have lower values of prism fusion range at distance (break and recovery of negative fusional vergence), as well as lower amplitudes of accommodation and reduced accommodative facility performances. Quaid and Simpson [17] demonstrated that children with reading difficulties not only have lower vergence facility results but are also more likely to have under-corrected hyperopia or overcorrected myopia. Furthermore, children with under-corrected hyperopia presented normal values of distance visual acuity, indicating that visual acuity alone is not a sufficient measurement of visual system functioning. Finally, Christian et al. [18] demonstrated that around six percent of children with reading difficulties have some sort of uncorrected refractive error, more than half of the children have distance and/or near fusional vergence results outside of norms, around twenty percent of children have a below-the-norm accommodative performance, and around one-third of children with reading difficulties have near phoria that falls outside of normal values. Uncorrected refractive errors can be linked to the presence of accommodative and binocular dysfunctions, including both non-strabismic and strabismic binocular dysfunctions. Dwyer and Wick [19] demonstrated that prescriptions of small corrections can significantly improve the performance of the accommodative and vergence systems. Furthermore, Scheiman and Wick [20] indicated that in all cases of accommodative, binocular, and ocular motor dysfunctions, the initial management consideration is the full correction of any significant refractive error.

Wilson et al. [21] and Klatte et al. [22] described that an intervention for struggling readers is most effective when involving teamwork between the family, speech and language therapists, teachers, psychologists, and other specialists, since reading difficulties might also be associated with different psychological, physiological, environmental, sociological, and linguistic factors. Furthermore, vision specialists might also play a role in improving a child’s academic performance by addressing the functioning of the visual system. While Bush et al. [23] showed a slight improvement in academic performance after vision therapy, the results regarding convergence insufficiency and reading performance pre- and post-therapy were inconclusive. However, the teacher reported a positive impression of vision therapy and its role in academic performance.

The role of optometrists remains unclear, as various studies have yielded inconclusive results regarding which visual functions may deviate from the norm in children with reading difficulties. Additionally, there is a lack of research on whether children with reading difficulties are more likely to exhibit various accommodative and binocular dysfunctions. Therefore, the current research aims to provide a comprehensive evaluation of subjective refraction, accommodation, and binocular functions in school-age children, both with and without reading difficulties, to enhance our understanding of the role of the visual system in academic performance.

## 2. Materials and Methods

### 2.1. Participants

Altogether, eighty-two children (6–12 years old, mean age 9.1 ± 1.9 years; 36 girls, 46 boys) from Marupe State Gymnasium participated in the study. All children acquired the general basic education and were not assigned to special education programs. The study was approved by the Life and Medical Sciences Research Ethics Committee of the University of Latvia (13/06/2022). Written informed consent of the parents or legal guardians of each child was obtained prior to the enrolment in the study.

All children were divided into two groups based on their reading performance: the target group (children with reading difficulties, N = 39, mean age 9.1 ± 1.8 years; 16 girls, 23 boys) and the control group (children without reading difficulties, N = 43, mean age 9.3 ± 2.0 years; 20 girls, 23 boys). Reading performance was assessed with the Acadience Reading (formerly DIBELS Next) test, adjusted and validated for the Latvian language [24]. Reading performance was assessed by professional speech therapists who are certified to perform the Acadience Reading test. Children in the target group were those whose Acadience Reading test results were below the 20th quartile, whereas children in the control group had results above the 20th percentile.

### 2.2. Method

All of the children were examined in the school setting during their school hours from 8:30AM to 2:00PM. Prior to the visual function examination, all children were systematically asked if they had any of the following complaints related to visual system performance: blurry vision at distance or near, diplopia at distance or near, difficulty focusing when switching distances, eyestrain, eye redness, eye pain or pulling sensation, light sensitivity, eye itching, burning, headache, visual fatigue, red or sore eyes [9,20].

After reviewing the case history and complaints, each participant underwent a full visual function examination, including objective refraction without cycloplegia (autorefractometer Huvitz HRK-1), uncorrected visual acuity (VA) at distance, full subjective sphero-cylindrical correction (using the monocular fogging technique for accommodation control [25]), and best corrected visual acuity (BVEA) at distance. In addition, all children were assessed for the following visual functions: near VA, amplitude of accommodation (push-up), dynamic retinoscopy (MEM), positive and negative relative accommodation (PRA and NRA), binocular and monocular accommodative facility (BAF and MAF, respectively; with ±2.00 D), vergence facility (if failed with 12 Δ BI/3 Δ BO, 8 Δ BI/8 Δ BO were applied), stereovision at distance (Osterberg test) and near (TNO test), suppression at distance and near (red filter test), angle of deviation with and without correction at distance and near (modified Thorington test), fusional reserves at distance and near (prism bar), and finally, near point of convergence (push-up method and dot card). Visual functions were determined with full subjective refraction at distance. All examinations (especially subjective refraction) were performed and controlled by certified optometrists. Some visual functions were assessed by 1st and 2nd year master’s students in optometry.

Since the management of accommodative and binocular dysfunctions should not be considered isolated from the refractive state, all children were analyzed for the presence of a significant refractive error and/or accommodative or binocular dysfunctions. The children were considered to have a significant refractive error if they demonstrated at least one of the following criteria: uncorrected VA at distance of 0.63 or less in decimal units (considering that the visual acuity demand for distance in modern primary school classrooms is 0.5 in decimal units) [26,27]; astigmatism of 0.75 D or higher; hyperopia level of 1.25 D or above [28,29]; uncorrected near VA lower than with correction. Accommodative and non-strabismic binocular dysfunctions were diagnosed based on the criteria presented by Franco et al. [30] and Scheiman and Wick [20], as well as updated criteria applied in other studies (see Table 1). Children presenting with one of the fundamental signs and at least two complementary signs were considered to have accommodative or non-strabismic binocular dysfunctions. The Cover test is the most useful test for detecting and characterizing strabismus. Since the Cover test allows for differentiation between heterotropia and heterophoria, it was performed on all children to determine whether the binocular dysfunction would be classified as non-strabismic or strabismic [31].

### 2.3. Statistical Analysis

Statistical associations between the reading performance and the amount of complaints as well as refraction, accommodative, and/or binocular vision disorders were determined using the Chi-square test of independence with a significance level of α = 0.05. Bar charts and tables depict the summary statistics.

## 3. Results

**Figure 1:**
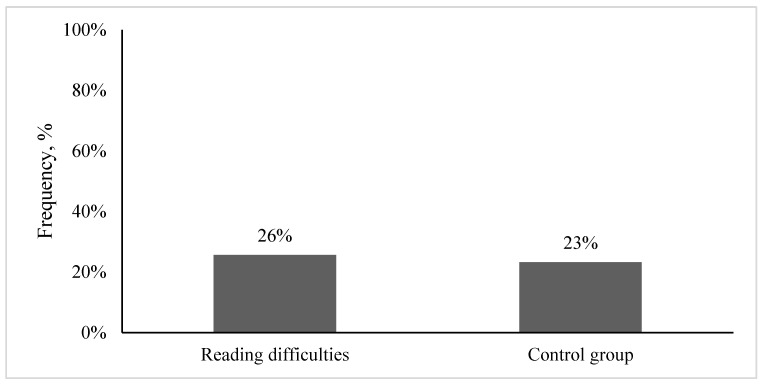
Frequency of visual complaints in children with reading difficulties and without reading difficulties (the control group).

The initial analysis of the complaints related to visual system performance demonstrated that 26% of children with reading difficulties and 23% of children without reading difficulties (the control group) presented with visual complaints (see Figure 1). Statistical analyses demonstrated that both groups presented with similar amounts of visual complaints (Chi-square test of independence: χ^2^ (1) = 0.063, *p* = 0.802, Cramer’s V/Phi = 0.802).

**Figure 2:**
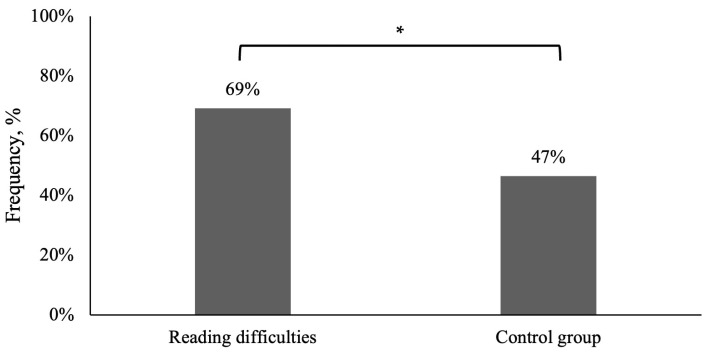
Frequency of significant refractive errors and/or accommodative and binocular dysfunctions in children with reading difficulties and without reading difficulties (the control group). * Statistically significantly difference (Chi-square test of independence *p* < 0.05).

However, both groups presented significant refractive errors and/or accommodative or binocular dysfunctions at least two times more often than visual complaints (see Figure 2), and the prevalence of dysfunctions was significantly higher in children with reading difficulties compared to the control group (Chi-square test of independence: χ^2^ (1) = 4.315, *p* = 0.038, Cramer’s V/Phi = 0.038; OR = 2.588, 95% CI 1.045–6.405).

**Figure 3:**
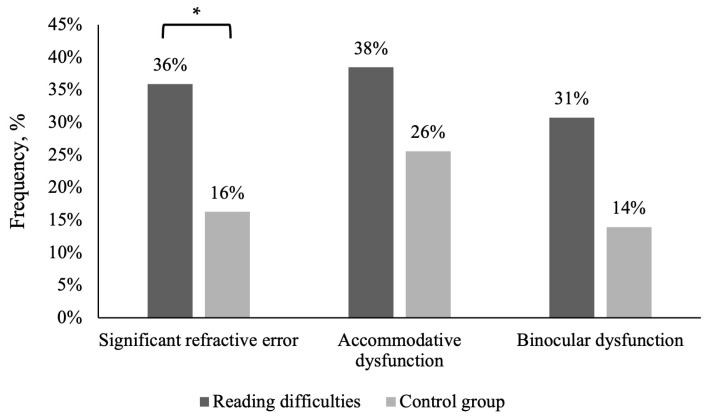
Relative frequency of significant refractive errors and accommodative and binocular dysfunctions in children with reading difficulties and without reading difficulties (the control group). * Statistically significantly difference (Chi-square test of independence *p* < 0.05).

In all children with significant refractive errors and/or accommodative or binocular dysfunctions, accommodative dysfunctions were diagnosed more often than binocular dysfunctions (see Figure 3). When addressed separately, the statistical analysis did not reveal a significant difference in the prevalence of accommodative dysfunctions (χ^2^ (1) = 1.567, *p* = 0.211, Cramer’s V/Phi = 0.211) or binocular dysfunctions (χ^2^ (1) = 3.375, *p* = 0.066, Cramer’s V/Phi = 0.066) between both groups. However, children with reading difficulties were more likely to present with a significant refractive error compared to the control group (χ^2^ (1) = 4.132, *p* = 0.042, Cramer’s V/Phi = 0.224).

Table 2 presents the relative frequency of the different types of accommodative and binocular dysfunctions (including both strabismic and non-strabismic) in both groups. The most common diagnosis was accommodative excess, which was combined with convergence insufficiency in some patients. The next most common diagnoses in children with reading difficulties were convergence excess and accommodative insufficiency, while ill-sustained accommodation was the next most common form in the control group.

## 4. Discussion

The present study explores visual functions in children with and without reading difficulties. The results demonstrate that around every fourth child presented with complaints that might be related to uncorrected refractive error and/or accommodative and non-strabismic binocular dysfunctions. A large amount of children will not complain but will still have an uncorrected refractive error and/or accommodative and binocular dysfunctions, with a significantly higher prevalence in children with reading difficulties. While one of the limitations of the current study is that a standardized questionary of visual complaints was not applied, all children were systematically asked if they had any of the most common complaints which were previously associated with refractive, accommodative, and binocular vision dysfunctions [9,20].

When delving into the self-perceived health of children, one must consider that there are different psychosocial factors that contribute to the evaluation of the child’s health by self-administered questionnaires [37]. Self-perceived health reports can be related to the child’s gender and even parental educational levels [38]. Dusek et al. [39] explored whether children with reading and writing difficulties were more likely to experience different visual or ocular complains. While in the study by Dusek et al. [39] children with reading and writing difficulties were more likely to complain of eye burning, tiredness after near-vision work, eye strain when looking at a near target, blurred vision, and diplopia, most of the complaints in this work were still similar in both groups (with and without reading and writing difficulties). The results of the current study only partly coincide with the results by Dusek et al. [39], which is more likely because the symptoms in the current study were not analyzed separately but in a generalized manner—whether there were or were not any complaints at all. Nevertheless, the current study demonstrates that the number of children with uncorrected refractive errors and/or accommodative and binocular dysfunctions was at least two times larger than the number of children with visual complaints, indicating that some of the dysfunctions might go unnoticed or undiagnosed due to the lack of noticeable nonvisual indicators (i.e., complaints). It leads to the question of what the necessary steps for parents, teachers, and primary care specialists are to understand whether the visual system is or is not interfering with a child’s daily life and learning process.

Visual complaints associated with uncorrected refractive error in children usually include blurry vision at distance or near, eyestrain, eye pain, watery or dry eyes, ocular pain, difficulty in recognizing faces, headache, glare, and eye irritation [9,40]. Cases of accommodative and binocular dysfunctions may also present with complaints of blurry vision at distance or near as well as diplopia at distance or near, difficulty focusing when changing fixation distances, eyestrain, eye redness, eye pain or pulling sensations, light sensitivity, eye itching, burning, headache, or covering one eye [20]. Teachers and parents have reported that they suspect that children could have uncorrected refractive error when they squeeze their eyes, have difficulties seeing the screen, and struggle copying information from the board, as well as having difficulties in reading or learning [40]. It is worth noting that many of these symptoms are not specific and can be related to other disorders as well, e.g., watery eyes and eye irritation can also be related to allergic reactions [41]; eye dryness, ocular irritation, and blurred vision might be related to dry eye syndrome [42]; and slow thinking, headaches, and reduced school performance might also be related to fatigue or even lack of sleep [43,44,45]. Therefore, even if a child reports specific complaints or parents and teachers ask specific questions to better understand the problem, the relation to a specific visual problem cannot always be established by evaluating the complaints of the child. Complaints serve as an important primary sign that the child needs a full comprehensive visual function examination by an optometrist.

The current study demonstrates that the prevalence of significant refractive error and/or accommodative and binocular dysfunctions in children without reading difficulties is around 47%, which is slightly higher than the results obtained by Ma et al. [46] and Lara et al. [35], which demonstrated that around 41.2% and 32.3% of children had normal binocular vision, respectively. Furthermore, the current study provides alarming results in children with reading difficulties: around two-thirds of these children presented with a significant uncorrected refractive error and/or accommodative and binocular dysfunctions. In addition, they presented more diversity in accommodative and binocular dysfunctions compared to children without reading difficulties. Since accommodative and binocular dysfunctions are often related to uncorrected refractive error, and different accommodative and binocular dysfunctions often co-exist, the current study aimed to explore the overall performance of the visual system. The data analysis included all of the inter-related factors and therefore led to a higher prevalence of significant refractive errors and/or accommodative and binocular dysfunctions.

As demonstrated by previous studies, children with reading difficulties can present with lower accommodative and vergence system performances, i.e., lower near visual acuity, larger exophoria, as well as lower accommodative facilities. However, the differences in the amplitude of accommodation and vergence facilities and the near point of convergence in children with and without reading difficulties still remain inconclusive [15,16,39,47]. Christian et al. [18] demonstrated that around 30% of children with reading difficulties had a near phoria and near point of convergence outside normal values, approximately 25% of children with reading difficulties had reduced stereoacuity, and 10% of children with reading difficulties had reduced binocular accommodative facilities. Furthermore, around 20% of these children required new spectacle correction. Cacho-Martínez et al. [9] demonstrated that some of the accommodative-system-related complaints could be resolved by correcting refractive error. Thus, all children with reading difficulties (with or without vision-related complaints) should undergo a very thorough assessment of refraction and visual function to ensure that any refractive and/or accommodative and non-strabismic binocular dysfunctions are prevented. Furthermore, even when a child has previously been diagnosed with accommodative or binocular dysfunctions, the child and their parents should be encouraged to perform regular check-ups of the child’s visual system performance. The current study revealed that two of the children with reading difficulties presented with previously diagnosed accommodative esotropia, and only one of them used their visual correction on a regular basis. 

To solve the observed vision problems in children with reading difficulties, the first and main step is always the optical correction of significant ametropia with spectacles or contact lenses. If an accommodative and/or binocular disorder is still diagnosed, next steps should consider different management options depending on the dysfunction [20], i.e., visual therapy, prism correction, and near additive. However, there is still an insufficient number of studies demonstrating the direct effects of such an approach on children’s reading skills. The existing studies also do not provide a clear answer about the importance of such an approach. While some studies only speculate that, from a physiological point of view, such an approach should play a major role in reading disorders, others demonstrate mixed results regarding the effects. Barrett [48] discussed that while accommodative and some non-strabismic binocular dysfunctions respond well to visual therapy, a generalized treatment for dyslexia patients that is not aimed at treating specific accommodative and binocular dysfunctions does not present an unequivocal effect. Handler and Fierson [49] indicated that vision disorders are rarely the primary cause of reading dysfunction and that visual correction or visual therapy only makes reading more comfortable but does not treat the main cause of reading difficulties. Further studies are necessary to explore what is the role of an appropriate visual correction and accommodative and non-strabismic binocular dysfunction management in children with reading difficulties.

Of all the children with reading difficulties involved in the current study, three participants were also diagnosed with unilateral amblyopia: one anisometropic amblyopia, one strabismic amblyopia (with partially accommodative esotropia and high degree hyperopia +6.00 D), and one combined amblyopia (with fully accommodative esotropia). While the binocular and accommodative functions of these children were improved by an appropriate refraction correction, only one of the three participants reported using refraction correction on a daily basis. Studies [50,51,52] have demonstrated that children with amblyopia can have a slower reading speed, which could be caused by reduced fixation stability and atypical saccadic eye movements [53,54,55]. Treatment for monocular amblyopia might not only improve vision quality but could also increase reading distance and improve reading speed and efficiency [50,56].

The current study was conducted in Latvia, where, based on the National Health Service guidelines for children’s healthcare, all children should have at least three visits at the ophthalmologist at the ages of 1–2, 3–5, and 6–7 years [57]. No other recommendations regarding eye health for older children are given, i.e., school-age children are not obligated to perform any vision screening, and the management of refractive errors and accommodative and binocular dysfunctions depends solely on the recognition of different symptoms by the child, parents, teachers, speech therapists, doctors, or other surrounding persons. While the informative material provided by the Children’s Clinical University Hospital in Latvia indicates that all children with reading difficulties should consult with an ophthalmologist in order to rule out any vision disorders, including functional disorders [58], different guidelines for dyslexia management point out that dyslexia is not related to visual system disorders [59]. The results of the current study do not imply that reading difficulties are caused by vision dysfunctions; rather, they suggest that each patient with reading difficulties should have a thorough visual system examination, since accommodative and binocular dysfunctions could potentially add a further hindrance to reading difficulties [60]. While the main limitation of the current study is the sample size, which is smaller than that applied in some other studies addressing the accommodative and binocular system performance in children with reading and learning difficulties [15,16,38], the comprehensive methodology applied in the current study not only allows for comparing the performance of different accommodative and vergence functions but also helps to determine the overall performance of the visual system. Therefore, it is one of only a few studies to explore the prevalence of accommodative and binocular dysfunctions in children with and without reading difficulties.

## 5. Conclusions

The current study demonstrated that children with reading difficulties are more likely to have either a significant refractive error or an accommodative and/or binocular vision disorder. In addition, children may not feel or even understand typical complaints associated with these disorders. While the observed prevalence of different accommodative and binocular dysfunctions is not the same as in other studies (most likely because different diagnostic criteria were applied), it can be noticed that children with reading difficulties presented with a higher variety of different accommodative and binocular dysfunctions than children without reading difficulties. Therefore, when working with children with reading difficulties, vision specialists should perform a full visual function examination even if the child does not present with any ocular or visual complaints. Additionally, even though reading is a multifactorial process and reading difficulties have been linked to different psychological, physiological, environmental, sociological, and linguistic factors, the improvement of the visual quality might also make at least a small contribution to facilitating the reading process by taking out at least one hindering factor and allowing the focus to be directed to other factors that might be contributing to the reduced reading performance.

## Figures and Tables

**Table 1:** Diagnostic criteria for accommodative and non-strabismic binocular dysfunctions (all diagnostic criteria are adapted from Franco et al. [30] and Scheiman and Wick [20]; for some accommodative and non-strabismic binocular dysfunctions, complementary signs were developed, indicated under the dysfunction type).

Dysfunction	Diagnostic Criteria
Accommodative insufficiency [32,33]	*Fundamental signs:* Reduced AA—at least 2.00 D less than the expected Hofstetter’s minimal value of the age and MAF < 6 cpm (difficulty with −2.00 D flipper) *Complementary signs:* BAF < 3 cpm (difficulty with −2.00 D flipper) MEM ≥ +0.75 D PRA ≤ 1.25 D PFV < 12/15/4 ∆—blur at near may be reduced
Ill-sustained accommodation	*Fundamental signs:* MAF < 6 cpm (difficulty with −2.00 D flipper) *Complementary signs:* BAF < 3 cpm (difficulty with −2.00 D flipper) MEM ≥ +0.75 D PRA ≤ 1.25 D PFV < 12/15/4 ∆—blur at near may be reduced
Accommodative excess [9]	*Fundamental signs:* MAF < 6 cpm (difficulty with +2.00 D flipper) *Complementary signs:* BAF < 6 cpm (difficulty with +2.00 D flipper) MEM ≤ +0.25 D NRA ≤ 1.50 D NFV < 9/17/8 ∆—blur at near may be reduced
Accommodative infacility [9]	*Fundamental signs:* MAF < 6 cpm (difficulty with ±2.00 D flipper) *Complementary signs:* BAF < 3 cpm (difficulty with ±2.00 D flipper) PRA ≤ 1.25 D NRA ≤ 1.50 D PFV < 12/15/4 ∆—blur at near may be reduced NFV < 9/17/8 ∆—blur at near may be reduced
Convergence insufficiency [34]	*Fundamental signs:* Near exophoria at least 4 ∆ larger than at distance *Complementary signs:* NPC > 6 cm AC/A ratio < 2:1 (the gradient method) PFV < 12/15/4 ∆ (reduced finings in at least one of three at near) VF < 13 cpm (difficulty with ∆ BO) BAF < 6 cpm (difficulty with +2.00 D flipper) NRA ≤ 1.50 D MEM ≤ +0.25 D
Convergence excess [35]	*Fundamental signs:* Near esophoria at least 2 ∆ larger than at distance *Complementary signs:* NFV < 9/17/8 ∆ (reduced findings in at least one of three at near) AC/A ratio > 6:1 MEM ≥ +0.75 D PRA ≤ 1.25 D VF < 13 cpm (difficulty with ∆ BI) BAF < 6 cpm (difficulty with −2.00 D flipper)
Fusional vergence dysfunction	*Fundamental signs:* PFV < 12/15/4 ∆ (reduced finings in at least one of three at near) or NFV < 9/17/8 ∆ (reduced finings in at least one of three at near) *Complementary signs:* VF < 13 cpm (difficulty with ∆ BO and BI) BAF < 6 cpm (difficulty with ±2.00 D flipper) PRA ≤ 1.25 D NRA ≤ 1.50 D
Divergence insufficiency [36]	*Fundamental signs:* Far esophoria at least 4 ∆ larger than at near *Complementary signs:* AC/A ratio < 2:1 NFV < -/4/2 ∆ (reduced finings in at least one of three at distance)
Divergence excess	*Fundamental signs:* Far exophoria at least 5 ∆ larger than at near *Complementary signs:* AC/A ratio > 6:1 PFV < 5/11/6 ∆ (reduced finings in at least one of three at distance) NFV < 9/17/8 ∆ (reduced finings in at least one of three at near)
Basic exophoria [9]	*Fundamental signs:* Significant exophoria at distance and near of approximately the same amount (within 5 ∆) *Complementary signs:* PFV < 5/11/6 ∆ (reduced finings in at least one of three at distance) PFV < 12/15/4 ∆ (reduced finings in at least one of three at near) VF < 13 cpm (difficulty with ∆ BO) BAF < 6 cpm (difficulty with +2.00 D flipper) NRA ≤ 1.50 D MEM ≤ +0.25 D
Basic esophoria [35]	*Fundamental signs:* Significant esophoria at distance and near of approximately the same amount (within 5 ∆) *Complementary signs:* NFV < -/4/2 ∆ (reduced finings in at least one of three at distance) NFV < 9/17/8 ∆ (reduced finings in at least one of three at near) VF < 13 cpm (difficulty with ∆ BI) BAF < 6 cpm (difficulty with −2.00 D flipper) PRA ≤ 1.25 D MEM ≥ +0.75 D

Abbreviations: AA—amplitude of accommodation (monocular); BAF—binocular accommodative facility; MAF—monocular accommodative facility; MEM—monocular estimated method of dynamic retinoscopy; PRA—positive relative accommodation; NRA—negative relative accommodation; PFV—positive fusional vergence; NFV—negative fusional vergence; NPC—near point of convergence; AC/A ratio—accommodative convergence against accommodation ratio; VF—vergence facility; BO—base out; BI—base in; PD—prism dioptres; D—dioptres; cpm—cycles per minute.

**Table 2:** The relative frequency (in percentage) of different accommodative and non-strabismic binocular dysfunctions in children with (*n* = 39) and without reading difficulties (*n* = 43). Some children had both types of dysfunctions (combination of accommodative and binocular dysfunctions).

Type of Dysfunction	Reading Difficulties N = 39	Control Group N = 43
Accommodative excess	21%	19%
Convergence excess	10%	2%
Convergence insufficiency	8%	7%
Accommodative insufficiency	8%	
Fusional vergence dysfunction	3%	2%
Basic exophoria	3%	2%
Basic esophoria	3%	2%
Ill-sustained accommodation	3%	7%
Accommodative infacility	3%	
Accommodative esotropia	5%	

## Data Availability

The data presented in this study are available on request from the corresponding author. The data are not publicly available due to privacy reasons.
